# CrossFit-Induced Rhabdomyolysis in a Young Healthy Indonesian Male

**DOI:** 10.7759/cureus.14723

**Published:** 2021-04-27

**Authors:** Sherly Lawrensia, Joshua Henrina, Alius Cahyadi

**Affiliations:** 1 Department of Medicine, Regional Public Hospital of Waikabubak, Nusa Tenggara Timur, IDN; 2 Department of Medicine, Balaraja Public Health Center, Tangerang, IDN; 3 Department of Internal Medicine, School of Medicine and Health Sciences, Atma Jaya Catholic University of Indonesia/Atma Jaya Hospital, Jakarta, IDN

**Keywords:** exercise-induced rhabdomyolysis, crossfit, strenuous exercise, overexcercise, rhabdomyolysis

## Abstract

CrossFit, a high-intensity interval training, keeps growing in trend and is one of the most favorable types of fitness, after wearable technology. However, an excessive workout is detrimental to the human body, particularly the muscle tissue. CrossFit is known to cause exercise-induced rhabdomyolysis, a concerning disease with potentially devastating consequences. Nevertheless, only a few case reports have described this disease, and this is the first case report of such disease in Indonesia to the best of our knowledge.

A 27-year-old, previously healthy and active Indonesian male presented with dark urine and myalgia in lower extremities after 720 repetitions of squats three days before admission. His urinalysis showed +1 blood, 2-7 red blood cells/high power field (HPF), but negative protein. Laboratory results showed an increase in creatine phosphokinase (CPK) (54,250 U/L) and LDH (1,670 U/L) levels, consistent with exercise-induced rhabdomyolysis, and serum calcium of 1.87 mmol/L, with normal serum creatinine and BUN level. He was hospitalized for two days and was treated with intravenous hydration therapy.

CrossFit-induced rhabdomyolysis is a potentially devastating disease. Apart from prompt diagnosis and treatment, further research regarding the safe number of repetitions for CrossFit training, particularly for lower extremities are needed. Predictors of CrossFit-induced rhabdomyolysis must be sought throughout, and participants’ awareness should be increased.

## Introduction

CrossFit, a high-intensity interval training, keeps growing in trend since it was established in 2000 by Greg Glassman, an ex-gymnast [1]. According to the Worldwide Survey of Fitness Trends of 2020, it was placed second in types of fitness favored after wearable technology [2]. It is well known that excessive workout is detrimental to the human body even to the healthiest, most experienced, and most well trained in doing strenuous exercise [3].

Rhabdomyolysis is the release of intracellular muscle cell components into the circulation due to strated muscle breakdown after skeletal muscle injury or necrosis [4-6]. Meanwhile, exercise-induced rhabdomyolysis (ER) is a process commenced with skeletal muscle breakdown, which occurs after doing strenuous exercise, and ultimately ends with the cell components being released into the systemic circulation, and gives rise to a multitude of signs and symptoms, and typical laboratory results [7].

The clinical manifestation of this disease is divided into local signs, such as muscle pain, skin bruises, tenderness, and muscle weakness, as well as systemic signs including dark-colored urine, as the first systemic sign, and sometimes accompanied by malaise and/or fever [5]. The dark-colored urine is attributed to the presence of myoglobin in the urine. The classical triad of rhabdomyolysis triad is dark-colored urine, muscle aches, and weakness. Unfortunately, this triad only exists in less than 10% of patients [8].

This disease is concerning and often results in hospitalization mandating IV fluid rehydration to prevent acute kidney injury and other subsequent complications [7]. In this article, we would like to report a case of rhabdomyolysis due to CrossFit developed in a previously healthy and active 27-year-old Indonesian male. To the best of our knowledge, this is the first case reporting such disease in Indonesia.

## Case presentation

A previously healthy and active 27-year-old Indonesian male, medical personnel, came to the emergency department (ED) with a chief complaint of a sudden change in urine color described as tea-colored urine. He also complained of soreness in both lower extremity muscles, which caused difficulty walking. He denied any recent trauma, but three days before he visited the ED, he had been doing CrossFit intensely. That was the first time of his workout after not doing any exercise or CrossFit session for the past three months.

His workout regimen consisted of 12 repetitions of 60 times overhead squats (720 repetitions in total), five repetitions of a one-minute duration of battle rope, wall ball, and kettlebell overhead, respectively, with a brief interval of rest in between. He denied any excessive fatigue during the exercise. Afterwards, he felt sore on both of his lower extremities, resulting in difficulty walking. He did not take any analgesics to alleviate his pain. His previous medical, family, and medication history was unremarkable.

His vital signs were within the normal limits (blood pressure of 120/80 mmHg, heart rate of 80 beats per minute, respiration of 16 cycles per minute, and axillary temperature of 36.5ºCelsius). The physical examination revealed lower extremities tenderness with light palpation without any bruises or swelling. Urinalysis showed +1 blood, 2-7 red blood cells/high power field (HPF), but negative protein (Table 1). Based on his basic metabolic panel, markers of skeletal muscle injury were increased and he was experiencing hypocalcemia. With the first day values for creatine phosphokinase (CPK) of 54,240 U/L (normal range: <190 U/L ), lactate dehydrogenase (LDH) of 1,670 U/L (normal range: <480 U/L), and serum calcium of 1.87 mmol/L (normal range: 2.2-2.7 mmol/L). However, his BUN (2.24 mmol/L; normal range: 2.1 to 7.1 mmol/L) and serum creatinine levels (70 mmol/L; normal range: 60-110 μmol/L) were normal (Table 1). His complete blood count was within normal limits.

Due to the history of massive exercise and rhabdomyolysis’ clinical presentations as well as increasing levels of CPK and LDH, the patient was diagnosed as ER. Subsequently, he was admitted to the internal medicine ward and received treatments consisting of the intravenous isotonic solution up to two litres for 24 hours and analgesic agents. He was also told to drink plenty of water (5-6 L/day).

The patient did not experience any other symptoms or deterioration during hospitalization. His urine output during the observation was at 2.4 mL/kg/h. On the third day of care, his urine color had reverted to normal and he was discharged from the hospital asymptomatic, without acute kidney injury or permanent physical damage. However, the CPK and LDH level were not checked on the discharge time. On the follow up after one week, his CPK value was 1,565 U/L, and the LDH level had decreased to the normal level (Table 1).

Based on a risk prediction score of in-hospital mortality or renal replacement therapy developed by McMahon et al. [9] with a score of 4, our patient was categorised under the low-risk category. However, this risk predictive score needs to be validated for its use outside tertiary hospitals and for use in other countries.

**Table 1:** Laboratory values during admission day until follow up BUN - blood urea nitrogen CPK - creatine phosphokinase LDH -  lactic dehydrogenase HPF - high power field

	Admission day	Day 2	Follow up (10 days after admission)
BUN (mmol/L)	2.24	NA	2.33
Creatinine (μmol/L)	70	NA	84.88
Sodium (mmol/L)	136	NA	139.7
Potassium (mmol/L)	3.57	NA	3.56
Chloride (mmol/L)	100.33	NA	102.34
Calcium (mmol/L)	1.87 (Low)	NA	2.44
Magnesium (mmol/L)	0.66 (Low)	NA	0.78
CPK (U/L)	54,240 (High)	NA	1,565 (High)
LDH (U/L)	1,670 (High)	NA	299
Urinalysis			
Color	Dark yellow	Light yellow	NA
pH	6.5	6.0	NA
Blood	+1	Negative	NA
Leukocyte Esterase	Negative	Negative	NA
Nitrite	Negative	Negative	NA
Leukocyte/HPF	0-3	Negative	NA
Erythrocyte/HPF	2-7	Negative	NA
Epithels/HPF	1-3	0-1	NA
Bacteria	Negative	Negative	NA
Crystal	Negative	Negative	NA
Fungal	Negative	Negative	NA

## Discussion

The popularity of high endurance weight training has been growing since these last two decades [3]. CrossFit, a high-intensity exercise training, requires strenuous and repetitive extremities movement consisting of aggressive weight-training regimens [7]. This intense exercise in an untrained person or even trained athletes can lead to ER [3]. However, CrossFit-induced rhabdomyolysis itself is not a new phenomenon. It has been described in several case reports and one case series [7,8]. Meyer et al. in San Francisco had reported a case of a previously healthy 31-year-old female with rhabdomyolysis after her first time Crossfit workout [7]. In their case, the patient developed ER after upper extremity exercises, without dark-colored urine, and her laboratory workup revealed an elevation in CPK with a value of 18,441 U/L.

Meanwhile, a study held by Hopkins et al. found a total of 11 patients with rhabdomyolysis among 523 patients with injuries following CrossFit activities [8]. Our patient’s characteristics and presenting symptoms were in line with their study patients, which occur in a 27-year-old male (34.9 ± 9.7 years), came with the chief complaint of dark urine (90.9%), with three days (2.9 ±1.5 days) of symptoms’ duration. Our case is also similar to the findings of a study by Kenney et al., which involved 499 military personnel. This study showed that ER mostly occurred in a young and healthy individual with a benign course of illness, in addition to higher CK levels in comparison to patients without ER [10].

Therefore, in order to establish the diagnosis of ER pertinent history taking, physical examination, and laboratory workup are crucial. A focused physical examination of the involved muscles, inspecting and palpating for any abnormalities such as muscle swelling, changes in skin color or sensation, and muscle strength is especially important. The definitive diagnosis of rhabdomyolysis is an increased plasma CPK level greater than five times the upper limit of normal [11]. However, using this biomarker as an indicator of ER proves to be challenging. In part, CPK level rises naturally after strenuous exercise, with the maximum value that has been reported of up to 10 times the upper limit of normal [8]. Nevertheless, a CPK level of more than 10,000 U/L is thought to be a threshold for diagnosing significant ER [12]. In addition, wide variations of increased CPK level and susceptibility exist. The same conditions and environment that cause ER in one subject may not necessarily cause ER in another subject.

Utilizing urine myoglobin as an indicator for ER might be useful. However, due to its rapid hepatic metabolism and elimination, it is proven to be difficult. On the contrary, CPK is more stable due to its slower elimination. Therefore, CPK is a more reliable biomarker for assessing the presence and intensity of muscle damage [11,13].

Our patient was treated successfully with intravenous fluid resuscitation. He had a complete resolution of symptoms and laboratory values without any sequelae, such as acute kidney injury or compartment syndrome. Currently, there are no fixed guidelines for rhabdomyolysis management. Nevertheless, because acute kidney injury is the most common complication, early oral rehydration and intravenous isotonic solution are the mainstays of rhabdomyolysis treatment in order to preserve renal function [5,14].

The isotonic fluid, such as 0.9% sodium chloride solution or normal saline, should be initially given in a large amount to prevent renal injury by clearing toxic substance and increasing renal perfusion pressure [7]. To date, there is still no published guideline regarding the specific amount of fluid that should be given, nevertheless, the urine output target should be maintained at 2 mL/kg/h or 200-300 mL per hour [4,7,8]. Potassium or lactate containing fluid should be avoided. In addition, observing the disease’s course, side effects, and urine output are also equally important. In our case, the patient received two liters of normal saline intravenously and drank six liters of mineral water each day. Of note, in asymptomatic hypocalcemia, as in our case, calcium correction is unnecessary because calcium ions deposited in the muscle will return to the extracellular space in the recovery phase [13].

Without prompt treatment, complications of rhabdomyolysis will ensue. Such complications include electrolyte disturbances (hyponatremia, hyperkalemia, hypocalcemia), acute kidney injury, and multiple organ failure. Prompt recognition of signs and symptoms complemented with proper treatments are crucial to prevent complications.

Several limitations exist in our case. Due to the relatively expensive cost, laboratory workup was not ordered daily during the patient's hospitalization. Second, the decision to discharge him on his third day of hospitalization relied on the overall clinical improvement, without data of CPK, LDH, and serum electrolytes. However, a follow-up laboratory workup was ordered seven days after he was discharged and had already normalized. Last, we did not check for urine myoglobin because unfortunately, that specific assay was unavailable.

Finally, the lack of reported cases regarding this disease in Indonesia might underscore the low awareness of CrossFit participants regarding this potentially devastating disease, or it might be under-reported. In addition to attenuate this deleterious outcome, an integrated approach that incorporates individual-specific monitoring, quantification, and regulation (i.e., training load management) may aid in decreasing this disease risk. Proper conditioning is mandatory before starting a strenuous exercise after a period of deconditioning to prevent the occurrence of ER. More importantly, we did not blame CrossFit as the sole cause of rhabdomyolysis. However, we highlight the importance of an appropriate workout regimen while exercising to prevent ER.

## Conclusions

CrossFit-induced rhabdomyolysis is a potentially devastating disease. Apart from prompt diagnosis and treatment, further research regarding the safe number of repetitions for CrossFit training, particularly for low extremities are needed. Moreover, predictors of CrossFit-induced rhabdomyolysis must be sought throughout, and participants' awareness should be increased.
